# Diagnosis of Smallpox

**Published:** 1909-08

**Authors:** 


### Diagnosis of Smallpox.

Dr. John M. Armstrong, in an article on "The Diagnosis of Smallpox" (*Archives of Diagnosis,* April, 1909), arrives at the following conclusions:

1. Except in rare instances and only in the presence of an epidemic is the positive diagnosis of smallpox justified before the appearance of the skin lesions.

2. The history of pre-eruptive illness serves only to confirm the diagnosis as made by the senses of sight and touch.

3. The smallpox papule has characteristics which make a positive diagnosis possible within a few hours after its appearance.

4. The papules appear first on the exposed parts, particularly on the forehead and the flexor surfaces of the wrists. They are under the epidermis, hard, round, flat-topped, umbilicated, rose-pink and waxy in appearance. All these characteristics are usually present.

5. In general the entire course of evolution of the lesion from papule, vesicle, pustule to scab formation is regular and characteristic.

6. The lesions vary in number. They may be few or so numerous as to become confluent, but the individual characters of the lesion are present in all cases.—*Memphis Medical.*

